# PRDM3 attenuates pancreatitis and pancreatic tumorigenesis by regulating inflammatory response

**DOI:** 10.1038/s41419-020-2371-x

**Published:** 2020-03-16

**Authors:** Jie Ye, Anpei Huang, Haitao Wang, Anni M. Y. Zhang, Xiaojun Huang, Qingping Lan, Tomohiko Sato, Susumu Goyama, Mineo Kurokawa, Chuxia Deng, Maike Sander, David F. Schaeffer, Wen Li, Janel L. Kopp, Ruiyu Xie

**Affiliations:** 1 Cancer Centre, Faculty of Health Sciences, University of Macau, 999078 Macau SAR, China; 2 Institute of Translational Medicine, Faculty of Health of Sciences, University of Macau, 999078 Macau SAR, China; 3 Laboratory of General Surgery, The First Affiliated Hospital, Sun Yat-sen University, 510275 Guangzhou, China; 4 Division of Medical Sciences, National Cancer Centre Singapore, Duke-NUS Medical School, Singapore, 169857 Singapore; 5 Department of Cellular and Physiological Sciences, University of British Columbia, Vancouver, BC V6T 1Z4 Canada; 6 Department of Hematology and Oncology, University of Tokyo, Tokyo, 113-8654 Japan; 7 Department of Pediatrics and Cellular and Molecular Medicine, University of California-San Diego, La Jolla, CA 92093 USA; 8 Department of Pathology and Laboratory Medicine, University of British Columbia, Vancouver, BC V6T 1Z4 Canada

**Keywords:** Pancreatic cancer, Pancreatitis

## Abstract

Pancreatic ductal adenocarcinoma (PDAC) is associated with metaplastic changes in the pancreas but the transcriptional program underlying these changes is incompletely understood. The zinc finger transcription factor, PRDM3, is lowly expressed in normal pancreatic acini and its expression increases during tumorigenesis. Although PRDM3 promotes proliferation and migration of PDAC cell lines, the role of PRDM3 during tumor initiation from pancreatic acinar cells in vivo is unclear. In this study, we showed that high levels of PRDM3 expression in human pancreas was associated with pancreatitis, and well-differentiated but not poorly differentiated carcinoma. We examined PRDM3 function in pancreatic acinar cells during tumor formation and pancreatitis by inactivating *Prdm3* using a conditional allele (*Ptf1a*^*CreER*^*;Prdm3*^*flox/flox*^ mice) in the context of oncogenic *Kras* expression and supraphysiological cerulein injections, respectively. In *Prdm3*-deficient mice, *Kras*^*G12D*^-driven preneoplastic lesions were more abundant and progressed to high-grade precancerous lesions more rapidly. This is consistent with our observations that low levels of PRDM3 in human PDAC was correlated significantly with poorer survival in patient. Moreover, loss of *Prdm3* in acinar cells elevated exocrine injury, enhanced immune cell activation and infiltration, and greatly increased acinar-to-ductal cell reprogramming upon cerulein-induced pancreatitis. Whole transcriptome analyses of *Prdm3* knockout acini revealed that pathways involved in inflammatory response and Hif-1 signaling were significantly upregulated in *Prdm3*-depleted acinar cells. Taken together, our results suggest that *Prdm3* favors the maintenance of acinar cell homeostasis through modulation of their response to inflammation and oncogenic *Kras* activation, and thus plays a previously unexpected suppressive role during PDAC initiation.

## Introduction

Pancreatic ductal adenocarcinoma (PDAC) is the most common malignancy in pancreas and the third leading cause of cancer-related deaths in US^1^. Emerging evidence suggests that pancreatic acinar cells can acquire ductal cell-like characteristics and downregulate genes maintaining acinar cell identify, also known as acinar-to-ductal metaplasia (ADM). This process is reversible because once the injury is resolved the acinar cell-derived ductal-like cells can revert back to acinar cells^2,3^. However, in the presence of additional stresses, such as a *Kras*^*G12D*^ mutation, ADM cannot be reversed and cells are “locked” into a transdifferentiated state before converting to precancerous pancreatic intraepithelial neoplasia (PanIN) lesions and subsequently invasive PDAC^4–6^. In mice, pancreatic tumorigenesis is dramatically hastened by the presence of pancreatitis^7,8^, while, in humans, induction of chronic inflammation is a common character in many known risk factors for pancreatic cancer including diabetes, pancreatitis, alcohol consumption and tobacco use^9^. However, the complete transcriptional program that regulates the interconversion of acinar cells to ductal-like cells and vice versa, and the role of these events in the context of tumorigenesis are still unclear.

PRDM3 is a nuclear transcription factor involved in many biological processes including hematopoiesis, development, cell differentiation and apoptosis^10^. PRDM3 belongs to the positive regulatory domain (PRDM) family proteins, which are characterized by an N-terminal PR (PRDI-BF1-RIZ1 homologous) domain followed by an array of C2H2 zinc finger motifs for sequence-specific DNA binding and a C-terminal binding protein (CtBP)-binding domain for protein-protein interactions^11^. PRDM3 is necessary for the maintenance of hematopoietic stem cells^12,13^. A recent study has reported that PRDM3 is weakly expressed in normal pancreatic acinar cells and upregulated in many PDAC precursor lesions and PDAC^14^. Using siRNA-mediated knockdown of PRDM3 in PK-8 pancreatic cancer cells, Tanaka and colleagues also showed that PRDM3 promotes pancreatic cancer cell proliferation and migration through the inhibition of a KRAS suppressor miR-96^14^. Despite this characterization of the effects of PRDM3 inhibition in pancreatic tumor cells ex vivo, the role of PRDM3 during tumor initiation from acinar cells in vivo is unclear.

Here, we used a CreER-inducible mouse model to genetically delete *Prdm3* specifically in adult acinar cells to examine the functional role of PRDM3 in pancreatic carcinogenesis. Our results show that *Prdm3* deficiency potentiates inflammation, promotes tumor initiation and dramatically accelerates malignant progression, which is consistent with our findings indicating that PRDM3 loss is significantly associated with poorer survival in patients with PDAC. We further demonstrate that PRDM3 is important to suppress the expression of genes involved in inflammatory response in pancreatic acinar cells. These findings suggest an inhibitory role of PRDM3 in pancreatic tumorigenesis. Future development of drugs that target PRDM3 might yield novel approaches to benefit the treatment of PDAC.

## Results

### PRDM3 is upregulated in pancreatitis, as well as well-differentiated PDAC and its high expression is associated with better survival in patients with PDAC

**Fig. 1:**
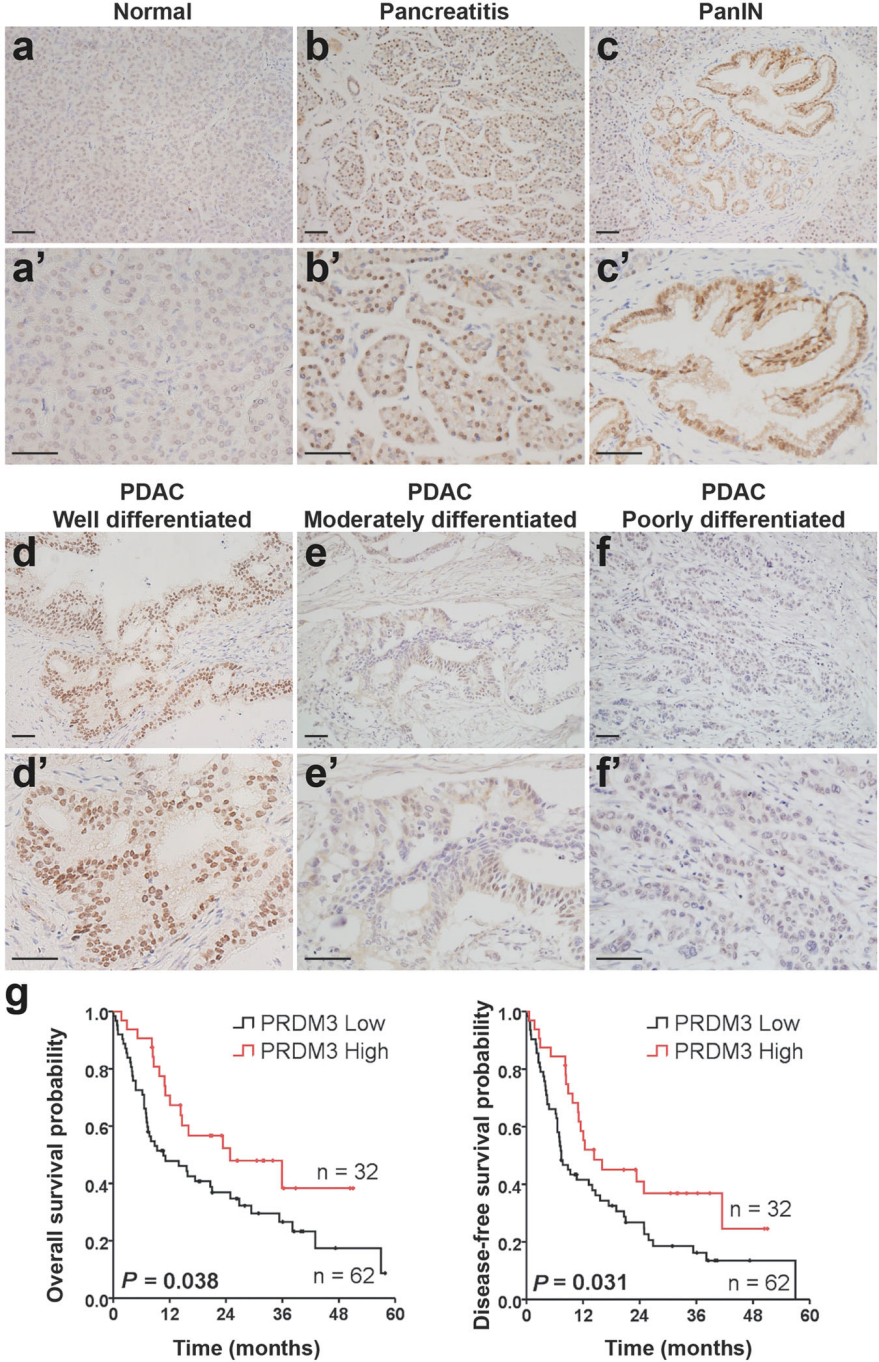
Weak PRDM3 expression is associated with poorly differentiated tumors and a worse prognostic outcome in patients with PDAC. Prdm3 immunostaining in normal pancreatic tissue (**a** and **a’**), pancreatitis (**b** and **b’**), PanIN (**c** and **c’**), well-differentiated (**d** and **d’**), moderately differentiated (**e** and **e’**), and poorly differentiated (**f** and **f’**) PDAC. **g** The overall survival probability and disease-free survival probability were compared between low (*n* = 32, staining index ≤ 6) and high (*n* = 62, staining index > 6) levels of PRDM3 expression in a cohort of 94 PDAC patients after surgical resection. *p*-values were calculated based on log-rank test. Scale: 50 μm.

We first characterized the expression of PRDM3 and its relevance to pancreatic cancer prognosis by analyzing a cohort of 94 patients who were diagnosed with PDAC and received surgical resection without preoperative chemotherapy. We found that PRDM3 was strongly expressed in precancerous PanIN lesions (Fig. 1c–c’) and well-differentiated PDAC from patients (Fig. 1d–d’), while moderately to poorly differentiated cancer cells showed little to no staining of PRDM3 (Fig. 1e–e’, f–f’, Table 1). Our observation of heterogeneous expression of PRDM3 in PDAC was supported by a recent study demonstrating that PRDM3 is selectively expressed in low-grade PDAC cells featuring differentiated epithelia, but not high-grade cells showing fibroblastoid morphology^15^. We performed subsequent overall survival and disease-free survival analyses with these 94 PDAC patients and found that patients with high levels of PRDM3 lived significantly longer than those with low levels of PRDM3 (Overall survival: 16.0 months vs. 9.3 months; Disease-free survival: 12.4 months vs. 7.4 months) (Fig. 1g). Our clinical relevance analysis clearly revealed that a better survival in patient with PDAC was associated with high levels of PRDM3 expression, but not with age, gender, tumor size, location, TNM (tumor-node-metastasis), or CA19-9 (Table 1). Given that pancreatitis is a well-described risk factor for PDAC development, we also analyzed pancreatic tissue from 22 patients with chronic pancreatitis. We found a dramatic increase of PRDM3 protein levels in inflamed tissues compared with normal pancreas (Fig. 1a–a’, b–b’; Supplementary Table 1). Similarly, administration of supraphysiologic concentrations of a cholecystokinin ortholog, cerulein, in mice resulted in acute pancreatitis and Prdm3 upregulation in murine acinar cells (Supplementary Fig. 1a). Consistent with findings from previous reports^14^, we also observed strong expression of Prdm3 in the precursor lesions of PDAC including ADM, low-grade PanINs, and high-grade PanINs found in pancreata from mice expressing oncogenic *Kras* in pancreatic acinar cells (*Ptf1a*^*CreER*^*; Kras*^*G12D*^) (Supplementary Fig. 1b). Together, our results demonstrated that elevated levels of PRDM3 are associated with inflamed pancreatic epithelia and well-differentiated pancreatic lesions, while low levels of PRDM3 are associated with poorly differentiated carcinoma and a worse prognostic outcome in patients with PDAC.

**Table 1:** Association between expression levels of PRDM3 and clinical relevance in patients with PDAC (*n* = 94).

Characteristics	Low expression (*n* = 62)	High expression (*n* = 32)	*p*-value^a^
Histological grade			**<0.001**
Poorly differentiated	19	1	
Moderately differentiated	43	14	
Well differentiated	0	17	
Survival^b^			**0.030**
Alive	17	16	
Death	45	16	
Age (years)			0.057
>60	24	19	
≤60	38	13	
Gender			0.174
Female	22	16	
Male	40	16	
Tumor size (cm)			0.300
≤2.0	10	8	
>2.0	52	24	
Location			0.333
Head	50	23	
Body and tail	12	9	
TNM^c^		0.717	
I, II	54	27	
III, IV	8	5	
CA19-9			0.526
≤35	12	8	
>35	50	24	

^a^*p*-values were based on *t*-test (two-sided). *p* < 0.05 was considered as statistically significant.

^b^All PDAC patients received surgical resection without preoperative chemotherapy; median follow-up was 12.1 months after surgical resection; median survival time for patients deceased was 8.0 months; median follow-up for patients alive was 26.4 months.

^c^The tumor-node-metastasis (TNM) stages were determined according to the 7th edition TNM classification of the American Joint Committee on Cancer.

Bold values indicate a statistically significant difference with a *p*-value less than 0.05.

### Ablation of *Prdm3* enhances Kras^G12D^-stimulated PDAC initiation and progression

To determine whether Prdm3 is functionally important for pancreatic carcinogenesis in vivo, we applied a genetic strategy to induce expression of oncogenic *Kras*^*G12D*^ and deletion of *Prdm3* in adult acinar cells, simultaneously. Cre-mediated recombination was induced in pancreatic acinar cells using the tamoxifen-inducible *Ptf1a*^*CreER*^ allele^16^. The *Mds1* and *Evi1* complex locus (*Mecom*) encodes a full-length isoform of *Prdm3*, and a shorter isoform lacking the N-terminus PR domain. We therefore used a *Prdm3*^*flox*^ mouse which harbors two *LoxP* sites flanking exon 4, the first shared exon in the long- and short-isoform of *Prdm3*^12^, to completely eliminate *Prdm3* in pancreatic acinar cells upon tamoxifen induced recombination. By combining the *Kras*^*LSL-G12D*^ allele^17^ with the *Ptf1a*^*CreER*^ allele with/without the *Prdm3*^*flox*^ allele, we generated control *Ptf1a*^*CreER*^*; Kras*^*G12D*^ (*Kras*^*G12D*^) mice, as well as *Ptf1a*^*CreER*^*; Kras*^*G12D*^*; Prdm3*^*flox/flox*^ (*Kras*^*G12D*^*-Prdm3*^*ΔAcinar*^) mice (Supplementary Fig. 2a).

**Fig. 2:**
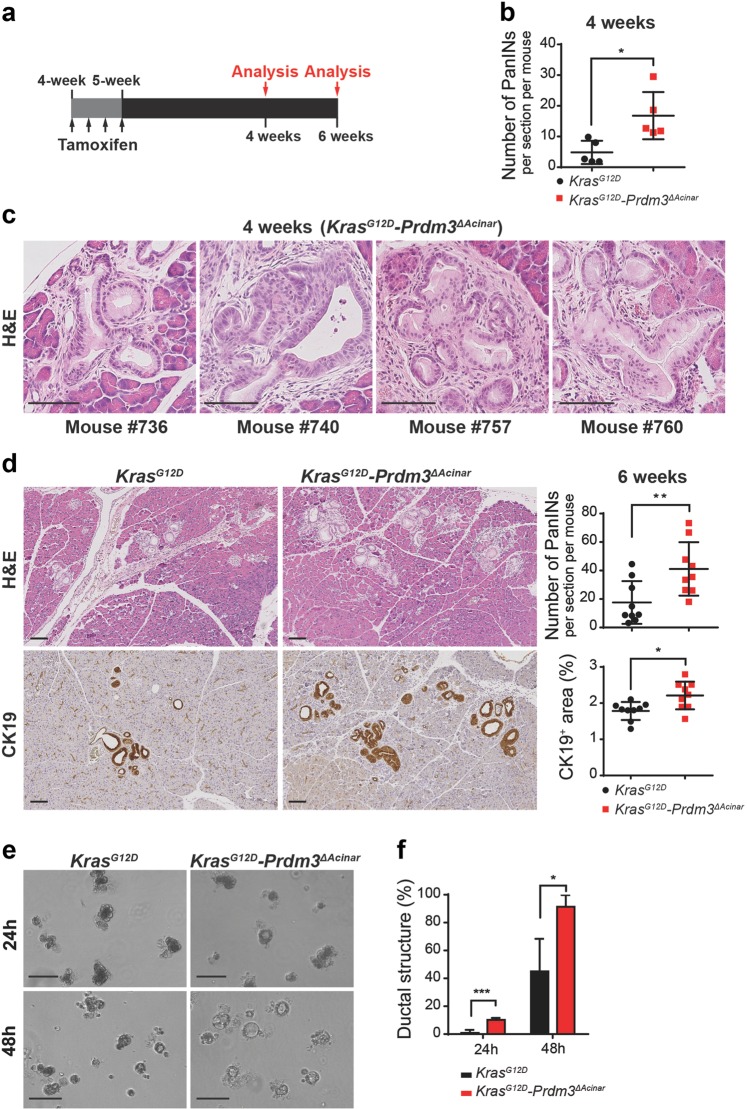
Loss of prdm3 promotes acinar-to-ductal metaplasia and PanIN lesions formation. **a** *Ptf1a*^*CreER*^*;Kras*^*G12D*^ and *Ptf1a*^*CreER*^*;Kras*^*G12D*^*;Prdm3*^*flox/flox*^ mice at 4–5 weeks of age were injected 4 times on alternating days with tamoxifen. Recombined mice were analyzed at 4 and 6 weeks post tamoxifen injection. **b** The number of PanINs per section for *Kras*^*G12D*^ (*n* = 5) vs. *Kras*^*G12D*^*-Prdm3*^*ΔAcinar*^ (*n* = 5) mice at 4 weeks post tamoxifen injection. **c** Representative images of high-grade PanINs in *Kras*^*G12D*^*-Prdm3*^*ΔAcinar*^ mice at 4 weeks after tamoxifen injection. **d** Hematoxylin-eosin staining (H&E) and immunohistochemistry staining for the ductal marker Cytokeratin 19 (CK19). Quantification of the number of PanINs, as well as the percent of pancreatic area that is CK19^+^ in *Kras*^*G12D*^ (*n* = 9) vs. *Kras*^*G12D*^*-Prdm3*^*ΔAcinar*^ (*n* = 9) mice 6 weeks post-tamoxifen injection. **e** Images of acinar cell explants embedded in Matrigel at 24 and 48 h. **f** Quantification of the percent of ductal-like structures in explants derived from *Kras*^*G12D*^ (*n* = 3) and *Kras*^*G12D*^*-Prdm3*^*ΔAcinar*^ (*n* = 3) mice at 4 weeks post-tamoxifen injection. Data show mean ± SD. Statistical analysis: Two-tailed *t*-test. *p < 0.05, **p < 0.01, ***p < 0.001. Scale: 100 μm.

To initiate recombination, we injected mice with tamoxifen at 4 to 5 weeks of age and analyzed pancreata at 4 weeks and 6 weeks post-injection (Fig. 2a). Comparison of *Prdm3* expression in *Kras*^*G12D*^*-Prdm3*^*ΔAcinar*^ and *Kras*^*G12D*^ mice after tamoxifen administration showed an almost complete loss of Prdm3 protein in *Kras*^*G12D*^*-Prdm3*^*ΔAcinar*^ acinar cells (Supplementary Fig. 2b, top panel). Four weeks after tamoxifen-mediated recombination, small areas of ADM and occasional low-grade PanINs were observed in the control *Kras*^*G12D*^ mice. In contrast, *Kras*^*G12D*^*-Prdm3*^*ΔAcinar*^ mice, with loss of *Prdm3* in pancreatic acinar cells, exhibited more cuboidal to columnar duct-like structures with enlarged lumens (Supplementary Fig. 2b, bottom panel). Quantification of the number of PanINs revealed that preneoplastic lesions arising in *Kras*^*G12D*^*-Prdm3*^*ΔAcinar*^ mice increased significantly compared to *Kras*^*G12D*^ mice (Fig. 2b). More intriguingly, we observed 4 out of 5 *Kras*^*G12D*^*-Prdm3*^*ΔAcinar*^ mice developed high-grade PanINs at 4 weeks post-tamoxifen injection (Fig. 2c), while no high-grade lesions were found in *Kras*^*G12D*^ mice even at 3 months of age (data not shown). Consistent with these findings, at 6 weeks post-tamoxifen injection *Kras*^*G12D*^*-Prdm3*^*ΔAcinar*^ mice exhibited higher number of lesions with histological and molecular characteristics of PanINs indicated by the expression of Cytokeratin 19 (CK19) (Fig. 2d) and the present of acidic mucin content indicated by Mucin 5AC (Muc5AC) and Alcian blue staining (Supplementary Fig. 2c). Altogether, these data suggest that deletion of *Prdm3* promotes PanIN formation in the presence of oncogenic *Kras* expression.

To further examine whether loss of *Prdm3* promotes acinar to ductal transformation in the presence of mutant Kras, we isolated primary acinar cell clusters from 8-week-old *Kras*^*G12D*^*-Prdm3*^*ΔAcinar*^ and *Kras*^*G12D*^ mice, respectively, and performed 3D Matrigel explant culture. In agreement with the histologic observations, *Prdm3* deficiency led to a significant increase in earlier ADM evens within 48 h in culture, as quantified by counting the number of duct-like structures vs. the total number of cell clusters (Fig. 2e, f). Previous studies have demonstrated that *Tgf-α* is upregulated in *Kras*^*G12D*^ pancreata^18^ and acts as a potent inducer for the transdifferentiation of acinar to ductal cells^19,20^. Therefore, we also tested if *Prdm3* depletion impacts TGF-α-induced in vitro ADM. Acinar cell explants derived from *Prdm3*^*ΔAcinar*^ mice transformed into duct-like structures within 30 h in culture with addition of TGF-α (Supplementary Fig. 2d, e). This acinar to ductal cyst conversion was almost undetected in explants derived from control (*Ptf1a*^*CreER*^) mice until 48 h in culture. Collectively, our data support that loss of *Prdm3* accelerates ductal metaplasia.

**Fig. 3:**
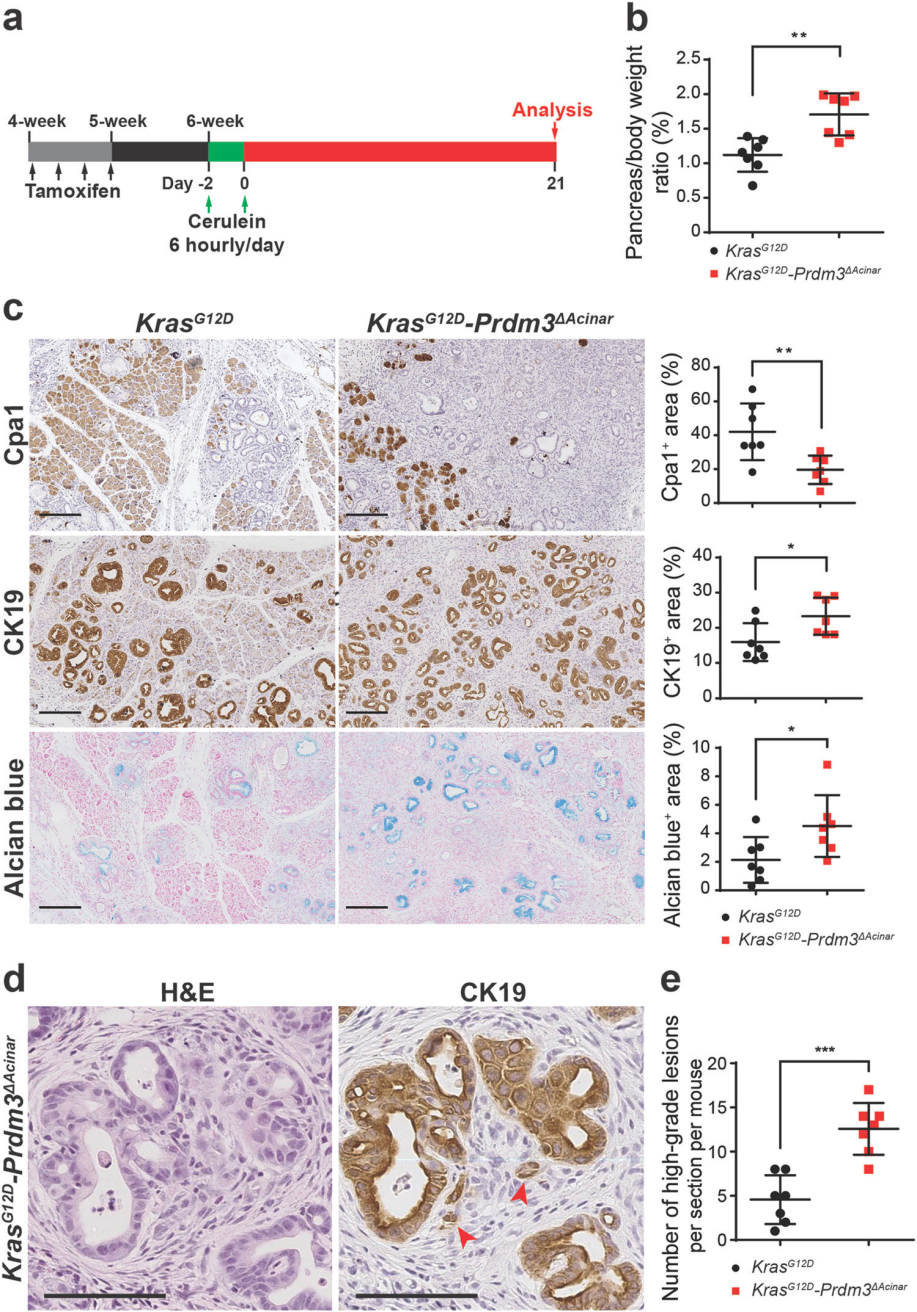
Inhibition of Prdm3 accelerates *Kras*^*G12D*^-driven neoplastic transformation in response to pancreatitis. **a** Schematic illustration showing experimental design of cerulein-induced acute pancreatitis in cooperation with activation of oncogenic *Kras* in *Ptf1a*-expressing cells. *Ptf1a*^*CreER*^*; Kras*^*G12D*^ (*n* = 7) and *Ptf1a*^*CreER*^*; Kras*^*G12D*^*; Prdm3*^*flox/flox*^ (*n* = 7) mice at 4 to 5 weeks of age were injected 4 times on alternating days with tamoxifen. One week after the last tamoxifen injection, mice were subjected to cerulein (50 μg/kg) injection at hourly intervals over 6 h on alternating days separated by 24 h and analyzed at 21 days post cerulein injection. **b** Quantification of relative pancreas mass measured as percent of pancreas weight over body weight in *Kras*^*G12D*^ vs. *Kras*^*G12D*^*-Prdm3*^*ΔAcinar*^ mice. **c** Hematoxylin-eosin staining (H&E) and immunohistochemistry for Cpa1, Cytokeratin 19 (CK19) and Alcian blue of pancreata from *Kras*^*G12D*^ and *Kras*^*G12D*^*-Prdm3*^*ΔAcinar*^ mice. Quantification of the percent of pancreatic area that is Cpa1^+^, CK19^+^ or Alcian blue^+^ in *Kras*^*G12D*^ vs. *Kras*^*G12D*^*-Prdm3*^*ΔAcinar*^ mice. **d** Representative images of tumor budding in *Kras*^*G12D*^*-Prdm3*^*ΔAcinar*^ mice indicated by red arrowheads. Immunohistochemistry staining of CK19 strongly suggests invasive high-grade neoplasia. **e** Number of high-grade PanINs per section for each genotype. Data show mean ± SD. Statistical analysis: Two-tailed *t*-test. *p < 0.05, **p < 0.01, ***p < 0.001. Scale: 200 μm (**c**) and 100 μm (**d**).

We next examined whether loss of *Prdm3* promotes neoplastic progression. To accelerate the formation of invasive lesions, we induced cerulein-mediated acute pancreatitis in cooperation with acinar-cell-specific activation of oncogenic *Kras* as previously described^4^. One week after tamoxifen administration, *Kras*^*G12D*^*-Prdm3*^*ΔAcinar*^ and *Kras*^*G12D*^ mice were injected hourly with 50 μg/kg cerulein over 6 h on alternating days. The pancreata were harvested at 21 days post-cerulein injection (Fig. 3a). Pancreata from *Kras*^*G12D*^*-Prdm3*^*ΔAcinar*^ mice had full spectrum of precursor lesions including low-grade PanINs, high-grade PanINs and ductal carcinoma in situ (Supplementary Fig. 3a), which had lost Prdm3 staining (Supplementary Fig. 3b). The control *Kras*^*G12D*^ mice had less evidence of tumorigenesis compared with *Kras*^*G12D*^*-Prdm3*^*ΔAcinar*^ mice. Specifically, in *Kras*^*G12D*^*-Prdm3*^*ΔAcinar*^ mice, the ratio of pancreas-to-body weight increased (Fig. 3b); the number of Cpa1^+^acinar cells decreased and CK19^+^ duct-like cells increased dramatically (Fig. 3c); a higher percentage of the pancreas was replaced by acidic mucin content indicated by Alcian blue staining (Fig. 3c); and the number of high-grade PanIN significantly elevated (Fig. 3e). Moreover, we consistently observed tumor budding associated with many high-grade neoplastic lesions in *Kras*^*G12D*^*-Prdm3*^*ΔAcinar*^ mice at 21 days post-cerulein injection (Fig. 3d). Tumor budding is a strong prognostic indicator of aggressive tumor behavior, which is defined as the presence of single cells or clusters of less than five tumor cells scattered in the stroma^21^. In contrast to *Kras*^*G12D*^*-Prdm3*^*ΔAcinar*^ mice, tumor budding was rarely observed in *Kras*^*G12D*^ mice, suggesting that loss of *Prdm3* accelerates pancreatic cancer formation in *Kras*^*G12D*^-expressing mice. Together, our data suggest that progression of low-grade precursor lesions to high-grade PanIN is more rapid in the absence of Prdm3.

### Loss of *Prdm3* in acinar cells enhances pancreatitis

**Fig. 4:**
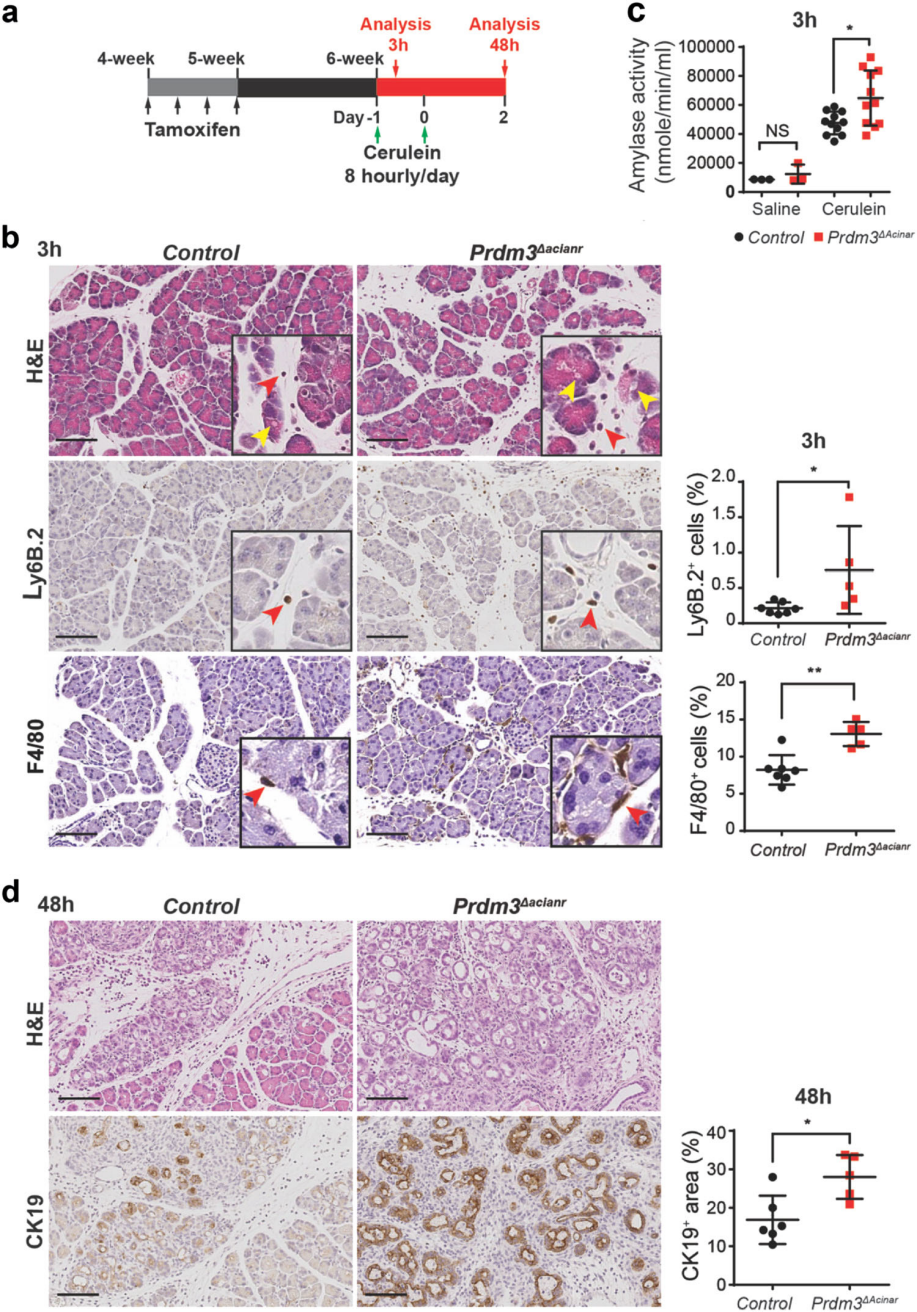
Prdm3 deletion in pancreatic acinar cells exaggerates cerulein-induced pancreatitis. **a** Schematic illustration of experimental design. *Ptf1a*^*CreER*^*;Prdm3*^*flox/flox*^ mice at 4 to 5 weeks of age were injected 4 times on alternating days with tamoxifen. One week after the last tamoxifen injection, mice were subjected to cerulein (50 μg/kg) injection at hourly intervals for 8 h per day for two consecutive days and analyzed at the indicated time points. **b** Histologic characterization was determined with hematoxylin-eosin staining (H&E). Immunohistochemistry staining for neutrophil marker Ly6B.2 and macrophage marker F4/80 with quantitation of the percent of all cells that are Ly6B.2^+^ or F4/80^+^ in *Ptf1a*^*CreER*^ (control, *n* = 7) and *Prdm3*^*ΔAcinar*^ (*n* = 5) pancreata. Vacuole is indicated by yellow arrowheads, and infiltrated immune cells are indicated by red arrowheads. **c** Serum amylase levels of cerulein-treated control (*n* = 11) vs. *Prdm3*^*ΔAcinar*^ (*n* = 11) mice and saline-treated control (n = 3) vs. *Prdm3*^*ΔAcinar*^ (*n* = 3). **d** Representative images of control and *Prdm3*^*ΔAcinar*^ pancreata stained with H&E and the ductal marker Cytokeratin 19 (CK19). Quantification of the respective percent of all cells that are CK19^+^ from control (*n* = 6) vs. *Prdm3*^*ΔAcinar*^ (*n* = 5) pancreata. Data show mean ± SD. Statistical analysis: Two-tailed *t*-test. *p < 0.05. Scale: 100 μm.

Given that inflammation promotes cancer formation and Prdm3 was significantly increased in humans with pancreatitis, we further determined whether Prdm3 modulated the inflammatory response of the pancreas in *Ptf1a*^*CreER*^*;Prdm3*^*flox/flox*^ mice. Mice injected with tamoxifen, but lacking the *Prdm3*^*flox*^ allele, were used as controls (*Ptf1a*^*CreER*^ mice). Seven days after tamoxifen administration Prdm3 protein was absent in more than 80% of acinar cells in *Prdm3*^*ΔAcinar*^ mice, indicating efficient and specific deletion of *Prdm3* (Supplementary Fig. 4a). Acute pancreatitis was induced with intraperitoneal injection of cerulein, as described previously^22^ (Fig. 4a). Specifically, 7 days after the last tamoxifen injection, *Prdm3*^*ΔAcinar*^ and control (*Ptf1a*^*CreER*^) mice were injected with 50 μg/kg cerulein at hourly intervals for 8 h. Histological assessment of pancreata from mice 3 h after the last cerulein injection demonstrated exaggerated interstitial edema, cytoplasmic vacuolization and immune cell infiltration in *Prdm3*^*ΔAcinar*^ mice compared with control mice (Fig. 4b). Consistently, examination of immune cell infiltration demonstrated a substantial increase in the number of F4/80^+^ macrophages, as well as Ly6B.2^+^ neutrophils in cerulein-treated *Prdm3*^*ΔAcinar*^ pancreata (Fig. 4b). Elevated blood amylase levels are an indicator of pancreatitis. Consistent with the increased inflammatory infiltrates in the pancreas, we observed significantly higher levels of serum amylase in *Prdm3*^*ΔAcinar*^ mice compared to controls (Fig. 4c). To determine whether the expression of inflammatory cytokines is increased, we harvested RNA from pancreata of *Prdm3*^*ΔAcinar*^ and control mice to perform quantitative qRT-PCR. As expected, expression of inflammatory cytokines, including *Il-6*, *Cxcl-1*, *Cxcl-10*, *Ccl2*, and *Ccl20*, increased significantly in *Prdm3*-deleted pancreata compared to control pancreata (Supplementary Fig. 4b). We further examined pancreata harvested from *Prdm3*^*ΔAcinar*^ and control mice 48 h after two series of 8-hourly injection of cerulein (Fig. 4a). Characterization by immunohistochemistry for the ductal marker CK19 confirmed that acini undergoing acinar-to-ductal metaplasia increased dramatically in *Prdm3*^*ΔAcinar*^ mice (Fig. 4d). These results illustrate that acinar-cell-specific ablation of *Prdm3* augments the severity of pancreatitis, suggesting a specific role of Prdm3 as a modulator of inflammatory response in the pancreas.

### Loss of *Prdm3* activates inflammatory response and Hif-1 signaling pathways

**Fig. 5:**
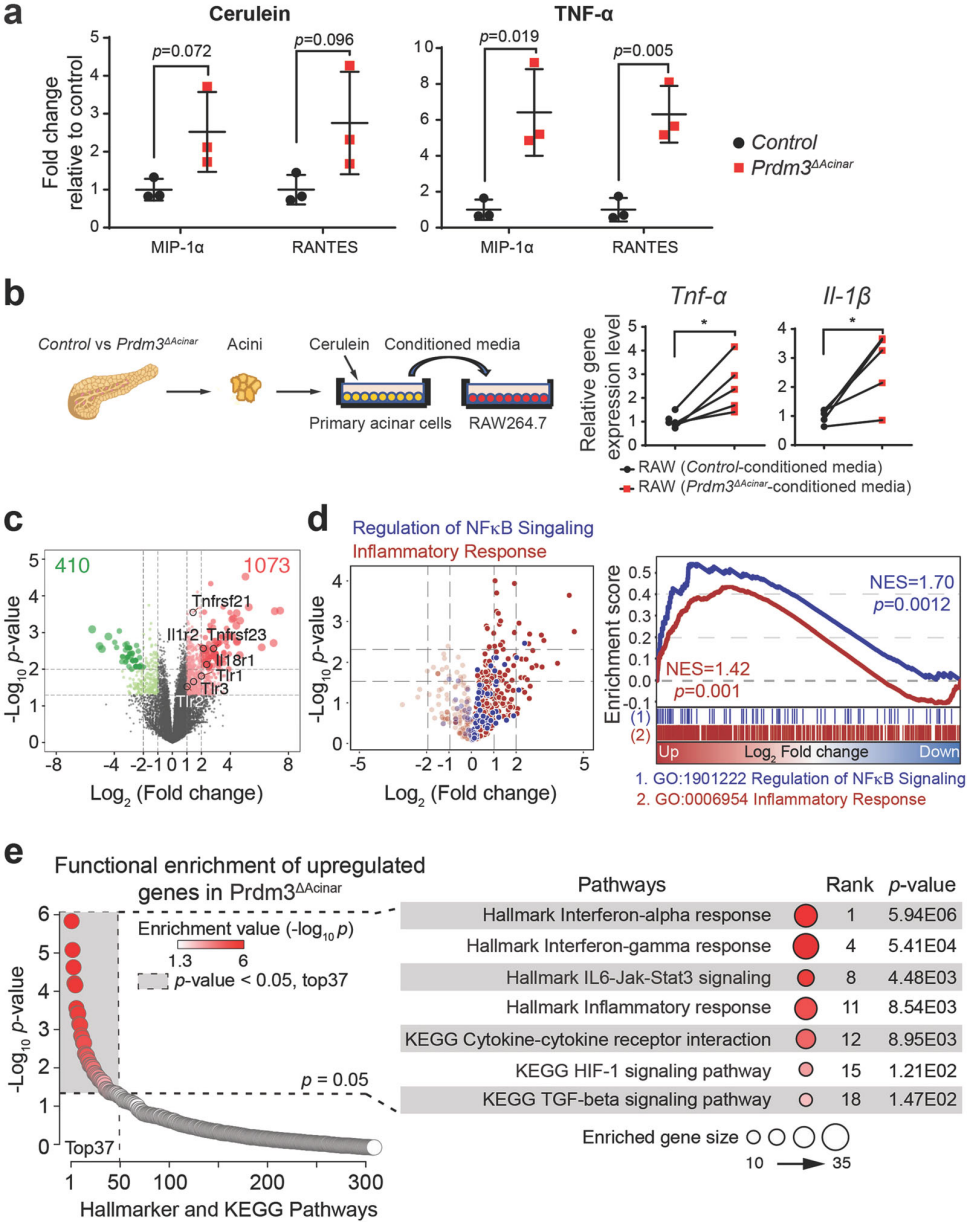
Inactivation of Prdm3 enhances inflammatory response pathways. **a** Cytokine secretion was determined using a Raybiotech mouse inflammation array. Primary acinar cells, isolated from control (*n* = 3) and *Prdm3*^*ΔAcinar*^ (*n* = 3) mice, were exposed to cerulein (10 nM) or TNF-α (100 ng/ml) for 6 h. Levels of cytokines (MIP-1α and RANTES) in culture medium were expressed relative to control. *p*-values were calculated based on two-tailed *t*-test. **b** Primary acinar cells were isolated from *Prf1a*^*CreER*^ (*n* = 5) vs. *Prdm3*^*ΔAcinar*^ (*n* = 5) mice and cultured in Waymouth complete media in the presence of 10 nM cerulein for 6 h. Raw264.7 cells were treated with the above acinar-cell-conditioned media for 16 h and collected for quantitative real-time PCR for the indicated cytokines. Each set of connected dots in **b** depicts an independent biological replicate (*n* = 5 experiments). Data show mean ± SD. Statistical analysis: Two-tailed *t*-test. *p < 0.05. **c** Volcano plot showing a total of 1483 differentially expressed genes in primary acinar cells isolated from *Prdm3*^*ΔAcinar*^ mice, relative to control *Ptf1a*^*CreER*^. Individual genes are labeled and circled in black. **d** Volcano plot showing differentially expressed genes belonging to inflammatory responses and NF-κB signaling are highlighted in red and blue, respectively. Gene Set Enrichment Analysis (GSEA) of differentially expressed genes (*Ptf1a*^*CreER*^ vs. *Prdm3*^*ΔAcinar*^) identified enrichment of immune responses and regulation of NF-κB signaling. Normalized enrichment score (NES) and *p*-values are shown. **e** Functional annotation on 1073 upregulated genes in primary acinar cells isolated from *Prdm3*^*ΔAcinar*^ mice, relative to control. Significant KEGG and Hallmark terms, *p*-values and ranks are shown.

Injured acinar cells initiate inflammatory responses by releasing digestive enzymes and proinflammatory mediators such as a variety of cytokines. These cytokines in turn attract and activate macrophages to produce excessive inflammatory cytokines, like tumor necrosis factor-alpha (TNF-*α*) and interleukin-1 beta (IL-1*β*), to exacerbate tissue injury^23,24^. To investigate whether loss of *Prdm3* alters cytokines secretion by pancreatic acinar cells, we performed a cytokine array assay on primary acinar cells in response to stimuli. Briefly, acinar cell clusters were isolated from a cohort of *Prdm3*^*ΔAcinar*^ and control (*Ptf1a*^*CreER*^) mice, respectively, and subsequently cultured in the presence of cerulein or TNF-*α* for 6 h. We found that among 40 cytokines detected in a mouse inflammatory array, macrophage inflammatory protein (MIP)-1α and regulated upon activation normal T expressed and secreted (RANTES) were remarkably increased in *Prdm3*^*ΔAcinar*^ incubation medium (Fig. 5a). As MIP-1α and RANTES are potent chemotactic agents for monocytes, we further examined macrophage activation in response to conditioned media from cerulein-stimulated cell cultures. The murine macrophages, Raw246.7 cells, were incubated in conditioned media collected from *Prdm3*^*ΔAcinar*^ or control cell culture exposed to cerulein for 6 h (Fig. 5b). After 16-hour incubation, the activation of macrophages was determined by the expression of inflammatory cytokines. We found that the relative expression of inflammatory cytokines *Tnf-α* and *Il-1β* was significantly higher in Raw246.7 incubated with *Prdm3*^*ΔAcinar*^ conditioned media (Fig. 5b), supporting that more proinflammatory factors were released from *Prdm3*-deficient cells to stimulate macrophage infiltration (Fig. 4b) and subsequent activation. These results indicate that *Prdm3*^*ΔAcinar*^ mice are more susceptible to cerulein-induced injury, at least, in part by contributing to increased macrophage activation.

To further examine the effect of *Prdm3* deletion on acinar cell homeostasis, we performed transcriptome analyses of primary acinar cells isolated from *Prdm3*^*ΔAcinar*^ and control mice. *Ptf1a*^*CreER*^*; Prdm3*^*flox/flox*^ and control *Ptf1a*^*CreER*^ mice were injected with four doses of tamoxifen at 4 to 5 weeks of age to induce recombination prior to acini isolation. One week after the last tamoxifen injection, primary acinar cells were isolated as described previously^25^ and then allowed to recover in oxygenated medium for 2 h. Total RNA was extracted from these isolated acini and subjected to RNA-seq analysis. The extent of *Prdm3* deletion was confirmed in *Prdm3*^*ΔAcinar*^ pancreata (Supplementary Fig. 5a). Analysis of RNA-seq data sets was performed by DESeq2. Setting a *p*-value threshold of 0.05, we identified 1483 genes that were significantly differentially expressed (log_2_|Fold change| > 1) in *Prdm3*-deficient acini compared with control (Supplementary Table 2). Moreover, 1073 out of 1483 differentially expressed genes (DEGs) were upregulated, while only 410 of them were downregulated (Fig. 5c). We found that the expression of many adhesion molecules involved in the inflammatory response, such as the TNF receptor superfamily *Tnfrsf21* and *Tnfrsf23*, interleukin receptors *Il18r1* and *Il1r2*, and toll like receptors *Tlr1*, *Tlr2* and *Tlr3* were significantly altered in *Prdm3*-deficient cells. Gene Set Enrichment Analysis (GSEA) further demonstrated that genes involved in inflammatory response and regulation of NF-κB signaling were significantly enriched in *Prdm3*-deficient acinar cells (Fig. 5d). These findings support that loss of *Prdm3* changes cellular homeostasis and biases acinar cells toward a proinflammatory state possibly by decreasing the inflammatory response threshold in *Prdm3*^*ΔAcinar*^ cells.

To further identify additional biological pathways that were activated upon *Prdm3* deletion, we performed functional annotation on the 1703 upregulated genes with KEGG and Hallmark data sets. Loss of *Prdm3* significantly affected over 30 pathways (Supplementary Table 3) including the Hif-1 signaling pathways (Fig. 5e). Intriguingly, recent studies demonstrated that mice with acinar cell-specific deletion of *Hif1α* were less susceptible to cerulein-induced pancreatitis^26^. As our transcriptome analysis revealed a significant elevation of *Hif1a* in *Prdm3*-deficient cells, we compared the protein level of Hif1a in control and *Prdm3*^*ΔAcinar*^ pancreata. We confirmed that the expression level of *Hif1a* was significantly upregulated in *Prdm3*-depleted pancreatic tissue by immunoblotting and immunohistochemical staining (Supplementary Fig. 5b, c). Our findings are supported by a previous study in which shRNA knockdown of *Prdm3* upregulated *Hif1a* in DA-1 and NFS-60 leukemic cells^27^. As loss of *Prdm3* exaggerated inflammation, we speculate that dysregulation of *Hif1a* expression might contribute to the dramatic effects of *Prdm3* depletion on pancreatitis.

## Discussion

In the present study, we investigated the role of *Prdm3* in pancreatitis and pancreatic tumorigenesis using mouse models to indelibly delete *Prdm3* in adult acinar cells. We demonstrated that PRDM3 was substantially upregulated in pancreatic acinar cells of patients with pancreatitis, as well as well-differentiated PDAC, but not poorly differentiated PDAC. Interestingly, our clinical relevance analysis suggests a prognostic relevance of low PRDM3 expression in PDAC patients with surgical resection. Consistent herewith, we further demonstrated that loss of *Prdm3* not only increased the severity of cerulein-induced pancreatitis, but also accelerated cellular atypia and tumorigenic potential in the pancreas, as *Prdm3*-deficient mice undergo robust formation of precursor lesions in the presence of oncogenic *Kras*. These findings implicate a potentially protective mechanism of *Prdm3* in pancreatic exocrine cells, which is different from the pro-tumor role of PRDM3 in several aggressive forms of cancer including colon, breast and ovarian cancer^28–30^. A number of alternatively spliced variants, including the long and short forms of *PRDM3*, are expressed in pancreatic cancer cells^14^. Our studies cannot distinguish which forms of PRDM3 are necessary for its anti-tumor effect in acinar cells, therefore, more work will be needed to examine the effects of different PRDM3 isoforms on pancreatitis and pancreatic cancer initiation.

Previous studies demonstrated that Prdm3 promotes proliferation and migration in established pancreatic cancer cell lines^14^. However, when *Prdm3* was knocked out in pancreatic acinar cells, we observed that *Prdm3* depletion potentiated pancreatic cancer initiation and progression to high-grade lesions including ductal carcinoma in situ, suggesting that Prdm3 plays a suppressive role in acinar-to-ductal transformation. At a molecular level, we found that over 70% of the differentially expressed genes between normal and *Prdm3*-deficient acinar cells were upregulated in *Prdm3*^*ΔAcinar*^ mice, which is consistent with previous studies demonstrating that Prdm3 acts as a transcriptional repressor through interaction with a variety of co-repressors, such as CtBP, histone methyltransferase SUV39H1 and deacetylase HDAC1/2^10^. We therefore postulate that increased Prdm3 expression in transformed ductal-like cells plays an inhibitory role in pancreatic tumorigenesis through the ability of Prdm3 to suppress a series of signaling cascades important for malignant transformation in exocrine pancreas.

Here, we showed that *Prdm3*-deficient acinar cells were much more susceptible to cerulein-induced injury. Loss of *Prdm3* enhanced ADM formation, as well as macrophage and neutrophil infiltration. Our transcriptome analysis suggests that deletion of *Prdm3* in adult pancreatic acini induces significant alteration in the expression profiles of cytokine/chemokine receptors, possibly accounting for greater responses to inflammatory stimuli. We speculate that accumulation of Prdm3 is beneficial to limit local inflammation by increasing the threshold for acinar cells to respond to cellular stress. Expression of oncogenic *Kras* in acinar cells triggers microinflammation and chemoattraction of macrophages^31^. Infiltrated macrophages secrete matrix-metalloproteinases and inflammatory cytokines, such as TNF-α, to facilitate acinar-to-ductal transdifferentiation through a NF-κB-mediated signaling cascase^32^. Moreover, several lines of evidence suggest that inflammation leads to an increase of Ras activity and amplification of oncogenic Ras signaling, which is necessary for pancreatic cancer progression^18,33,34^. Activation of *Kras* alone in mice leads to PDAC formation at a low frequency and takes over one year^35^. In contrast, when pancreatitis was induced in *Kras*^*G12D*^ mice, tumorigenesis occurred within a few months^8,36^ suggesting that precancerous lesions can arise from acinar cells through a process dramatically hastened by inflammation^32^. In this study, we demonstrated that loss of *Prdm3* accelerated *Kras*^*G12D*^-induced PanIN initiation and promoted rapid progression of pre-neoplastic lesions to invasive lesions. Given that activation of inflammatory response elevates constitutive Ras activity, we propose that, loss of *Prdm3* upregulates the expression of genes involved in inflammatory response in pancreatic acinar cells, which modulates acinar cell homeostasis to lower the threshold of acinar cells to inflammatory stimuli and promote widespread formation of precancerous lesions from *Kras* mutated acinar cells.

In addition, we found that hypoxia inducible factor *Hif1a* was significantly upregulated in *Prdm3*-deficent acinar cells at both mRNA and protein levels. Our findings are consistent with the previous study in which knockdown of Prdm3 upregulated *Hif1a* in DA-1 and NFS-60 leukemic cells^27^. It has also recently been established that HIF-1 signaling plays important roles in both pancreatitis and pancreatic cancer. *Hif1a* is overexpressed in chronic pancreatitis^37^ and its high expression is associated with poor prognosis in PDAC^38,39^. Acinar-cell-specific deletion of *Hif1a* prevented intrapancreatic coagulation of fibrinogen and protected mice from cerulein-induced acute pancreatitis^26^, suggesting a functional role of Hif1a in the development of pancreatitis. Given that loss of *Prdm3* exaggerated inflammation, we speculate that dysregulation of *Hif1a* expression might contribute to the dramatic effects of *Prdm3* depletion on pancreatitis.

Taken together, our data demonstrated that loss of *Prdm3* not only increased the severity of cerulein-induced pancreatitis, but also accelerated cellular atypia and tumorigenic potential in the pancreas, as *Prdm3*-deficient mice undergo robust formation of precursor lesions in the presence of oncogenic *Kras*. We uncovered a previously unappreciated role for Prdm3 as a suppressor of both pancreatitis and pancreatic tumorigenesis presumably through regulating inflammatory and Hif-1 signaling pathways in the pancreatic acinar cells.

## Materials and methods

### Human samples

A total of 94 patients diagnosed with PDAC and 22 patients diagnosed with chronic pancreatitis between 2003–2011 were included in this study in accordance with institutional guidelines and approved by the Clinical Research Ethics Committee of the First Affiliated Hospital at Sun Yat-sen University. Written informed consent was received from participants prior to inclusion in this study.

### Mice

All animal experiments were approved by the University of Macau Animal Ethics Committees and carried out in accordance to recommendations stated in the Guide for the Care and Use of Laboratory Animals for the National Institutes of Health (US Department of Health, Education, and Welfare). *Prdm3*^*flox/flox*^, *Kras*^*LSL-G12D*^, and *Ptf1a*^*CreER*^ mice have previously been described. Recombination was induced by four subcutaneous injection of tamoxifen every other day at 125 mg/kg body weight on animals at 4 to 5 weeks of age.

### Cerulein-induced pancreatitis

To induce acute pancreatitis, experimental mice were fasted overnight before administration of cerulein as described previously^4^. Cerulein (American Peptide) was dissolved in saline and administrated intraperitoneally at 50 μg/kg body weight hourly for 8 h. Mice were sacrificed after 3 h recovery. Alternatively, mice were injected with cerulein (50 μg/kg body weight) at hourly intervals for 8 h per day for two consecutive days and sacrificed at 48 h after the last injection. To accelerate tumorigenesis, mice were given hourly injections of cerulein (50 μg/kg body weight) for 6 h per day on alternating days separated by 24 h and sacrificed after 21 days.

### Histology and immunohistochemical analyses

Paraffin-embedded sections were prepared and subjected to hematoxylin, eosin Y, Alcian blue or immunohistochemical staining. H&E staining and IHC followed our established procedures^5^, including antigen retrieval with citrate buffer (pH 6.0) prior to staining paraffin sections. For Alcian blue staining, paraffin sections were incubated in 3% acetic acid for 3 min, followed by staining in 1% Alcian blue staining solution for 30 min, and subsequently in Nuclear Fast Red for 5 min. All slides were scanned with a 20× objective using a 2D glass slide digital scanner (Leica Biosystems) and examined at high magnification using the Aperio ImageScope software (Leica Biosystems). The Aperio positive pixel Algorithm was used to quantify area with positive staining and Aperio nuclear V9 algorithm was used to quantify the number of nuclei. The percentages of Cpa1-positive, CK19-positive, Muc5AC-positive, and Alcian blue-positive area were calculated by positive pixels divided by the total pixels in selected tissue areas. The percentages of Ly6B.2-positive, and Prdm3-positive cells were calculated by positive number of nuclei divided by the total number of nuclei in selected tissue areas. Six sections, which displayed maximal pancreatic cross-sectional area, from each animal were used for quantification. The number of PanINs were counted based on the characteristics of every gland with cuboidal to columnar duct-like structures and enlarged lumen in six sections per mouse. For the number of high-grade lesions, including PanINs and ductal carcinoma in situ, five 1600 × 920 μm squares in one section per mouse were analyzed and the squares were randomly distributed in the pancreas. Primary and secondary antibodies used for staining is provided in Supplementary Table 4.

### Evaluation of immunostaining of PRDM3 in patient specimens

PRDM3 expression was evaluated according to the staining intensity and proportion of positively stained tumor cells. Staining intensity was graded as 0 (negative), 1 (weakly positive), 2 (moderately positive), and 3 (strong positively). The proportion of positively stained tumor cells was scored as 0 (no positive cells), 1 (<10% positive cells), 2 (10–25% positive cells), 3 (25–50% positive cells), and 4 (>50% positive cells). The immunostaining of PRDM3 was determined by staining index (SI) through multiplying the staining intensity by the proportion of positively stained tumor cells as previously described^40^. The expression levels of PRDM3 was regarded as high if the SI score is > 6, or low if the SI score if ≤ 6. The immunohistochemical specimens were evaluated by two independently pathologists who were blinded to clinical diagnosis.

### RNA isolation and quantitative real-time PCR

Mice were sacrificed with CO_2_ asphyxiation followed by cervical dislocation. Pancreata were immediately harvested and cut into small pieces in RNALater (Qiagen). To detect mRNAs, 20 mg of tissue was homogenized in 1 ml of Trizol using a T 10 basic ULTRA-TURRAX® homogenizer. RNA was extracted from Trizol according to the manufacturer’s instructions (Thermo Fisher Scientific) and subsequently subjected to reverse transcription using the PrimeScript™ RT reagent Kit (TaKara). Quantitative real-time PCR was performed with the Premix Ex Taq (TaKara) on a CFX96 qPCR system (BioRad). Results were normalized to *Gapdh* for mRNA detection. The quantitative real-time PCR primer sequences are list in Supplementary Table 5.

### Western blotting

Pancreata were harvested and snap frozen in liquid nitrogen. The frozen tissue was homogenized in lysis buffer containing 20 mM Tris-HCl (pH 7.5), 150 mM NaCl, 1 mM Na_2_EDTA, 2 mM Na_2_VO_4_, 1% Triton X-100, 5 mM 4-nitrophenyl phosphate, 0.5% sodium deoxylcholate, 1 mM phenylmethanesulfonylfluoride, and protease inhibitor cocktail (Sigma). 20 μg of protein was separated on a 10% sodium dodecyl sulfate-polyacrylamide gel, transferred onto a polyvinylidene difluoride membrane, and probed with antibodies. The membranes were visualized using the ECL™ Western Blotting Detection System (GE Healthcare) and ChemiDoc™ Imaging Systems (BioRad).

### Serum amylase assay

Blood was collected by cardiac puncture and placed at room temperature for 30 min. Serum was separated from red blood cells by centrifugation at 2500 × *g* for 15 min. The top layer which contained serum was transferred to a new tube for further analysis. Amylase activity was measured using the Amylase Colorimetric Assay Kit (Sigma) following manufacturer’s instructions.

### Isolation of primary acinar cells

Primary acinar cells were isolated as described in detail previously^25^ with a small modification. In brief, pancreas was harvested and transferred into ice-cold Hank’s balanced salt solution (HBSS). Lymph nodes, fat and mesenteric tissues were carefully removed. Pancreas was minced into 2-mm to 3-mm pieces and digested with 0.2 mg/ml collagenase P (Roche) at 37 °C for 10–12 min. Cell clusters were washed 3 times with ice-cold HBSS containing 5% FBS and filtered through 100-μm cell strainer (BD Biosciences). The cell suspension containing acini was carefully layered on top of HBSS containing 30% FBS. Primary acinar cells were pelleted (80 × g, 2 min, at 4 °C) and resuspended in Waymouth media (Sigma).

### RAW246.7 cell culture and activation

Raw264.7 macrophages were obtained from American Type Culture Collection (ATCC) and maintained in DMEM (Gibco) containing 10% FBS and 100 U/ml penicillin/streptomycin in a 37 °C humified incubator supplemented with 5% CO_2_. To stimulate macrophage activation, 5 × 10^5^ Raw264.7 cells per well of 24-well plate were culture in DMEM (10% FBS and 100 U/ml penicillin/streptomycin) overnight and then incubated in acinar-cell-conditioned media. To obtain acinar-cell-conditioned media, primary acinar cell clusters isolated from an equal mass of *Prdm3*^*ΔAcinar*^ or control pancreata were incubated in Waymouth complete media (10% FBS, 10 mM HEPES, 100 U/ml penicillin/streptomycin, 100 μg/ml Soybean Trypsin Inhibitor, and 1 μg/ml Dexamethasone) containing 10 nM cerulein for 6 h. 0.5 ml cell-free supernatants were collected and applied to Raw264.7 cells for 16 h. RNA from Raw264.7 macrophages was extracted for quantitative real-time PCR. Data were normalized to *Gapdh*, as well as the amount of proteins extracted from primary acinar cells used to prepare conditioned media.

### Mouse cytokine array

Six-week-old recombinant *Ptf1a*^*CreER*^*;Prdm3*^*flox/flox*^ and *Ptf1a*^*CreER*^ mice were sacrificed one week after the last tamoxifen injection. Primary acinar cells were isolated from an equal mass of *Prdm3*^*ΔAcinar*^ (*n* = 3) and control (*n* = 3) pancreata as described above. To stimulate cytokine release, acinar cell explants were incubated in 0.5 ml Waymouth complete medium in the presence of cerulein (10 nM) or TNFα (100 ng/ml) for 6 h. 100 μl of supernatants was collected to examine the concentration of 40 cytokines using the Quantibody® Mouse Inflammatory Array Kit (RayBiotech, Catalog number QAM-INF-1) according to the manufacturer’s instructions. The fluorescent signal was detected using an InnoScan® 300 Microarray Scanner and analyzed by the GenePix® Microarray Analysis Software.

### 3D acinar cell explant culture

Three-dimensional Matrigel explant culture of acinar cells was performed as described previously^41^. In brief, freshly isolated primary acinar cell clusters were prepared as above, embedded in growth factor reduced Matrigel (BD Biosciences), cultured in Waymouth complete medium, and maintained at 37 °C in 5% CO_2_ atmosphere. To stimulate acinar to ductal transdifferentiation in the absence of mutant *Kras*, control and *Prdm3*^*ΔAcinar*^ acinar explants were treated with 50 ng/ml TGF-α. The ratio of acinar to ductal conversion was determined with an average of ten random 10× fields using the EVOS FL Imaging System (Thermo Fisher Scientific).

### RNA-seq analyses

Total RNA was extracted from primary acinar cells freshly isolated from 6-week-old *Ptf1a*^*CreER*^*;Prdm3*^*flox/flox*^ mice (*n* = 2) and their corollary controls (*Ptf1a*^*CreER*^, *n* = 2), one week after the last tamoxifen injection (125 mg/kg, 4 times, every other day). RNA concentration and integrity were measured using the Agilent 2100 Bioanalyzer (Agilent Technologies). PolyA tailed RNA were selected using Dynabeads® oligo(dT) (Thermo Fisher Scientific). cDNA libraries were prepared using the NEBNext® Ultra™ RNA Library Prep Kit for Illumina (New England Biolabs) according to the manufacturer’s instructions. Libraries were sequenced at Novogene (Tianjin, China) with 100× coverage and 150 bp paired end reads on an Illumina HiSeq 2500 instrument.

The quality of the sequencing data was analyzed by using FastQC (version 0.11.5), and raw reads with low quality were removed using Trim Galore (version 0.4.4) prior to analysis of the data. All the trimmed reads were mapped to reference mouse genome (mm10, GRCm38) by using STAR (version 020201), and the mapped counts were extracted using feature count from Subread package (version 1.5.3). Subsequently, read count data containing 49,492 quantified transcripts with raw reads was preprocessed by filtering out genes with zero read count across different samples, and 20,069 genes remained after filtering. The read count data were normalized by DESeq2, which can be used for downstream differential expression analysis. Differentially expressed genes (DEG) in *Prdm3*^*ΔAcinar*^ vs. control were filtered by |log_2_ fold change| > 1. *P*-values were determined using moderated *t*-statistics implemented in the Limma package. Gene set enrichment analysis (GSEA) was performed using Bioconductor R package clusterProfiler^42,43^. The gene sets implemented were derived from Cancer hallmark, Kyoto Encyclopedia of Genes and Genomes (KEGG) and Gene Ontology (GO), which was collected in the Molecular Signatures Database (MSigDB; version 6.2). Functional enrichment was performed on genes upregulated Prdm3-deficient cells. RNA-seq raw data generated for this manuscript is available in the NCBI Sequence Read Archive (SRA) accession number PRJNA605571.

### Statistical analysis

All data are presented as mean ± SD from at least three mice in each experimental group. Statistical analysis between two groups was acquired using two-tailed Student’s *t*-test. Statistical analyses of patient samples were performed with SPSS software using two-sided *t*-test. Kaplan-Meier survival plots were generated using the log-rank test. Statistical analysis for RNA-seq data is described in the corresponding sections. *P*-value < 0.05 was considered statically significant.

## Supplementary information

Supplementary information

Supplementary Figure 1

Supplementary Figure 2

Supplementary Figure 3

Supplementary Figure 4

Supplementary Figure 5

Supplementary Table 1

Supplementary Table 2

Supplementary Table 3

Supplementary Table 4

Supplementary Table 5
