# Supplementary material for: PRDM3 attenuates pancreatitis and pancreatic tumorigenesis by regulating inflammatory response
Source: Cell Death Dis. 2020 Mar 16;11(3):187. doi: 10.1038/s41419-020-2371-x (PMC7075911; doi:10.1038/s41419-020-2371-x)
Supplement: Supplementary file 1 — Supplementary information [file 41419_2020_2371_MOESM1_ESM.docx]

**Supplementary materials**

**Supplementary Fig. 1.** **Elevated PRDM3 expression is associated with inflamed pancreatic epithelia as well as premalignant pancreatic malignant lesions. (a)** C57BL/6 mice were injected with cerulein (50 μg/kg, 8 hourly per day, 2 consecutive days) to induce acute pancreatitis. Pancreata were harvested 48 hours after the last cerulein injection and subjected for hematoxylin-eosin staining (H&E) and Prdm3 immunostaining. **(b)** Immunohistochemistry for Prdm3 and H&E of ADM, low-grade PanIN and high-grade PanIN of *Ptf1a^CreER^;Kras^G12D^* pancreata. Scale: 100 μm

**Supplementary Fig. 2.** **Generation of Prdm3 conditional knockout mouse. (a)** Strategy to generate Prdm3 conditional knockout mice: *Ptf1a^CreER^;Kras^G12D^;Prdm3^flox/flox^* mice. Mice were injected with tamoxifen (TM) at 4-5 weeks of age and pancreata were analyzed at 4 and 6 weeks after the last tamoxifen injection. **(b)** Immunohistochemistry for Prdm3 and hematoxylin-eosin staining (H&E) in *Kras^G12D^-Prdm3^ΔAcinar^* and *Kras^G12D^* pancreata 4 weeks post-tamoxifen injection. **(c)** Immunohistochemistry for Muc5AC and Alcian blue staining of pancreata from *Kras^G12D^* and *Kras^G12D^-Prdm3^ΔAcinar^* mice at 6 weeks post-tamoxifen injection. Quantification of the percent of pancreatic area that is Muc5AC^+^ or Alcian blue^+^ in *Kras^G12D^* (n=9) vs. *Kras^G12D^-Prdm3^ΔAcinar^* (n=9) mice. **(d)** Images of acinar cell explants embedded in Matrigel at 30 and 48 hours. **(e)** Quantification of the percent of ductal-like structures in explants derived from *Prdm3^ΔAcinar^* (n=5) and *Ptf1a^CreER^* (control, n=5) mice in culture with addition of TGF-α (50 ng/ml) for 30 and 48 hours. Data show mean ± SD. Statistical analysis: Two-tailed *t*-test. **p* < 0.05, ***p* < 0.01. Scale: 100 μm.

**Supplementary Fig. 3.** **Full spectrum of pancreatic precursor lesions in Prdm3-deficient mice.** **(a)** Hematoxylin-eosin staining of representative whole-section and high-magnification images of *Kras^G12D^-Prdm3^ΔAcinar^* and *Kras^G12D^* pancreata 21 days post-cerulein injection. Low-grade PanINs are indicated by stars, high-grade PanINs are indicated by arrowheads, and ductal carcinoma *in situ* is indicated by arrow. **(b)** Immunohistochemistry for Prdm3 in *Kras^G12D^-Prdm3^ΔAcinar^* and *Kras^G12D^* mice. Scale: 3 mm (a top) and 100 μm (a bottom and b).

**Supplementary Fig. 4.** **Prdm3-deleted mice are more susceptible to cerulein-induced inflammatory responses. (a)** *Ptf1a^CreER^* control and *Ptf1a^CreER^;Prdm3^flox/flox^* mice were injected with tamoxifen at 4-5 weeks of age and pancreata were analyzed at 7 days after the last tamoxifen injection. Hematoxylin-eosin staining (H&E) and immunohistochemistry for Prdm3. **(b)** Quantitative real-time PCR for indicated cytokines in control (n=7) vs *Prdm3^ΔAcinar^* (n=5) pancreata from mice 3 hours after injection of cerulein 8 times at hourly intervals. Data show mean ± SD. Statistical analysis: Two-tailed *t*-test. **p* < 0.05, ***p* < 0.01, *** *p* < 0.001. Scale: 100 μm.

**Supplementary Fig. 5.** **Immunostaining reveals an upregulation of Hif1α in *Prdm3^ΔAcinar^* pancreata. (a)** Quantitative real-time PCR for *Prdm3* in primary acinar cells isolated from *Prdm3^ΔAcinar^* (n=3) and control pancreata (n=3). **(b)** Hif1α immunoblot analysis of pancreas lysates from *Ptf1a^CreER^* control and *Prdm3^ΔAcinar^* mice. Equal loading was confirmed by immunoblot with an anti-α-tubulin antibody. Densitometric analysis of Hif1α normalized to α-tubulin is shown on the right. **(c)** Immunohistochemistry for Hif1α of *Prdm3^ΔAcinar^* and *Ptf1a^CreER^* control pancreata. Data show mean ± SD. Statistical analysis: Two-tailed *t*-test. **p* < 0.05, **p* <0.01. Scale: 100 μm

**Supplementary Table 1. PRDM3 expression in normal and pancreatitis tissue**

**Supplementary Table 2.** Excel spreadsheet indicates differential expressed genes (| Log_2_FC | > 1, *p* < 0.05) in Prdm3-depleted pancreatic acinar cells.

**Supplementary Table 3.** Hallmark and KEGG pathway analyses of upregulated genes in Prdm3-depleted pancreatic acinar cells. Excel spreadsheet shows the output pathways, *p*-values, gene ratio, relative gene counts and gene IDs.

**Supplementary Table 4. List of primary and secondary antibodies.**

**Supplementary Table 5. List of primers for quantitative real time-PCR.**
