# Supplementary material for: PRDM3 attenuates pancreatitis and pancreatic tumorigenesis by regulating inflammatory response
Source: Cell Death Dis. 2020 Mar 16;11(3):187. doi: 10.1038/s41419-020-2371-x (PMC7075911; doi:10.1038/s41419-020-2371-x)
Supplement: Supplementary file 7 — Supplementary Table 1 [file 41419_2020_2371_MOESM7_ESM.docx]

**Supplementary Table 1.**

| **PRDM3 expression in normal and pancreatitis tissue** | |
| --- | --- |
| **Histology** | **Staining intensity score (0 / 1 / 2 / 3)** |
| Normal (n=8) | 1 / 6 / 1 / 0 |
| Pancreatitis (n=22) | 0 / 3 / 4 / 15 |
