# Supplementary material for: PRDM3 attenuates pancreatitis and pancreatic tumorigenesis by regulating inflammatory response
Source: Cell Death Dis. 2020 Mar 16;11(3):187. doi: 10.1038/s41419-020-2371-x (PMC7075911; doi:10.1038/s41419-020-2371-x)
Supplement: Supplementary file 10 — Supplementary Table 4 [file 41419_2020_2371_MOESM10_ESM.docx]

**Supplementary Table 4.**

| **Primary antibody** | | | |
| --- | --- | --- | --- |
| **Antigen** | **Species** | **Source** | **Dilution** |
| Prdm3 | Rabbit | Cell signaling | 1:500 |
| Ly6B.2 | Rat | Serotec | 1:1000 |
| Cytokeratin 19 | Rabbit | Abcam | 1:2000 |
| Muc5AC | Mouse | Thermo Scientific | 1:500 |
| Cpa1 | Goat | R&D | 1:1000 |
| Hif1α | Rabbit | NOVUS | 1:500 |
| α-Tubulin | Mouse | Santa Cruz | 1:1000 |
| F4/80 | Rat | Abcam | 1:500 |
| **Secondary antibody** | **Conjugation** | **Source** | **Dilution** |
| Rabbit/Rat/Mouse/Goat | Biotinylated | Vector Laboratories | 1:500 |
| Rabbit/Mouse | HRP | Jackson Immunoresearch | 1:2000 |
