# Supplementary material for: PRDM3 attenuates pancreatitis and pancreatic tumorigenesis by regulating inflammatory response
Source: Cell Death Dis. 2020 Mar 16;11(3):187. doi: 10.1038/s41419-020-2371-x (PMC7075911; doi:10.1038/s41419-020-2371-x)
Supplement: Supplementary file 11 — Supplementary Table 5 [file 41419_2020_2371_MOESM11_ESM.docx]

**Supplementary Table 5.**

| **Primers for quantitative real time-PCR** | | |
| --- | --- | --- |
| *Il-6* Fw | ATGAACAACGATGATGCA | 127bp |
| *Il-6* Rv | CCAGAAGACCAGAGGAAA |  |
| *Cxcl-1* Fw | CTGGGATTCACCTCAAGAACATC | 117bp |
| *Cxcl-1* Rv | CAGGGTCAAGGCAAGCCTC |  |
| *Cxcl-10* Fw | GCACGAACTTAACCACCATCTTCC | 179bp |
| *Cxcl-10* Rv | CTACCCATTGATACATACTTGATGACAC |  |
| *Ccl2* Fw | GGCTCAGCCAGATGCAGTTA | 185bp |
| *Ccl2* Rv | GGACCCATTCCTTCTTGGGG |  |
| *Ccl20* Fw | CCTGATTTGTGTCCCAGTGGACTT | 188bp |
| Ccl20 Rv | TTAATCCTTCCACTAAGCGCCC |  |
| *Il-1β* Fw | TGACGGACCCCAAAAGAT | 122bp |
| *Il-1β* Rv | GTGATACTGCCTGCCTGA |  |
| *Tnf-α* Fw | CCAAAGGGATGAGAAGTTCC | 133bp |
| *Tnf-α* Rv | CTCCACTTGGTGGTTTGCTA |  |
| *Gapdh* Fw | GTGTTCCTACCCCCAATGTGT | 248bp |
| *Gapdh* Rv | ATTGTCATACCAGGAAATGAGCTT |  |
